# Promoting Clinical Breast Evaluations in a Lower Middle–Income Country Setting: An Approach Toward Achieving a Sustainable Breast Health Program

**DOI:** 10.1200/JGO.17.00103

**Published:** 2018-01-25

**Authors:** Roziya Buribekova, Irina Shukurbekova, Surayo Ilnazarova, Nekruz Jamshevov, Guldarbogh Sadonshoeva, Saleem Sayani, Aliya Aminmuhammad, Farin Amersi, Sheemain Asaria, Mansoor Saleh, Zohray Talib

**Affiliations:** **Roziya Buribekova**, **Irina Shukurbekova**, **Surayo Ilnazarova**, **Nekruz Jamshevov**, and **Guldarbogh Sadonshoeva**, Aga Khan Health Services, Dushanbe, Tajikistan; **Saleem Sayani** and **Aliya Aminmuhammad**, Aga Khan University; **Farin Amersi**, **Sheemain Asaria**, **Mansoor Saleh**, and **Zohray Talib**, Aga Khan Health Board, Karachi, Pakistan; **Zohray Talib**, George Washington University School of Medicine, Washington, DC; and **Mansoor Saleh**, University of Alabama at Birmingham Comprehensive Cancer Center, Birmingham, AL.

## Abstract

**Purpose:**

To promote a systems-based approach for the early detection and downstaging of breast cancer at presentation in the remote mountainous region of Gorno Badakhshan Autonomous Oblast (GBAO), Tajikistan, by introducing breast cancer awareness into the community and training health care professionals in clinical breast evaluation (CBE).

**Methods:**

Through a public-private partnership between the Ministry of Health, the Aga Khan Health Services, Tajikistan, and the Aga Khan Health Board, we organized breast cancer screening in the community and trained family medicine doctors (FMDs) and family medicine nurses (FMNs) in CBE. We identified and trained CBE master trainers, who, in turn, systematically trained FMNs to conduct CBEs in each of the remote regional clinics.

**Results:**

Between 2014 and 2017, 47 FMDs (85% of all FMDs in GBAO), 166 FMNs (55% of all FMNs in GBAO), and six master trainers were trained. Of 3,556 women who were screened, abnormal CBEs were noted in 696 of them (20%). Of the last 1,101 CBEs that were performed by trainee FMNs, with secondary CBEs by master trainers, the rate of abnormal CBEs plateaued at 9%. A total of 18 women were diagnosed with breast cancer—2.6% of abnormal CBEs and 0.5% of all screened women.

**Conclusion:**

A dual-pronged approach of community awareness and CBE training of health care professionals, supported by CBE master trainers, offers a sustainable approach for the early detection of breast pathology. We observed anecdotal evidence of clinical early-stage detection over time with improved CBE proficiency and community acceptance. Sustaining this program will require advocacy by health care providers and a responsive public policy to support the early detection and treatment of breast cancer across the region.

## INTRODUCTION

Breast cancer remains the number one cause of female mortality in lower middle–income countries (LMICs).^1^ Whereas a lack of adequate diagnostic and therapeutic resources has been identified as a major hurdle in the optimal delivery of breast cancer care, delayed diagnosis continues to be an area of significant concern. Lack of awareness about breast cancer in the population, lack of health care professionals who are trained in clinical breast examination (CBE), and inadequate diagnostic and treatment resources contribute to a delay in medical intervention and treatment.^2^

The rates of early detection and improved survival observed in at upper middle–income countries (UMICs)^3^ cannot be readily replicated in LMICs. In contrast, training of health care professionals in CBE and the incorporation of CBE into the routine clinical evaluation of adult female patients represents an opportunity for the early identification and clinical downstaging of breast cancer at the time of presentation.^4^

**Fig 1:**
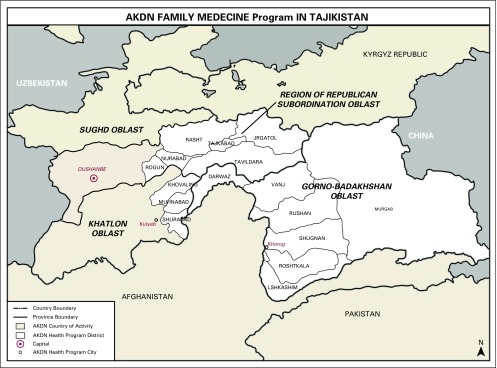
Breast cancer program initiative in the Department of Health of the Autonomous Oblast of Gorno Badakshan. AKDN, Aga Khan Development Network.

We embarked on a multidisciplinary approach to introduce context-relevant comprehensive breast health in an LMIC setting. In Gorno Badakhshan, we observed a close-knit community with a well-organized infrastructure to support health care. A public-private partnership between the Aga Khan Health Services, Tajikistan (AKHSTj), and the Department of Health of Gorno Badakshan Autonomous Oblast (GBAO) has resulted in a functional, three-tiered delivery of health care in this isolated mountainous region in the South East of Tajikistan. Primary tier 1 basic care at the village level is provided by trained family medicine nurses (FMNs) who provide health education and low-level maternity/postpartum and basic screening services, secondary tier 2 care at the district clinic is provided by trained family medicine doctors (FMDs), and tertiary tier 3 specialized care is provided at the Oblast capital in Khorugh, with subspecialty referrals to the federal capital in Dushanbe (Fig 1).

Between 2014 and 2017, volunteer clinicians from the Aga Khan Health Board (AKHB-USA) collaborated with AKHSTj to introduce breast cancer awareness in GBAO, with a focus on developing a sustainable program of CBE and helping to improve diagnostic and therapeutic services dedicated to breast cancer. The inaugural AKHB-USA team consisted of a multidisciplinary team of clinicians, surgeons, radiologists, and pathologists who established a framework for breast cancer awareness and multidisciplinary management.^5^

Here, we present the outcome of these efforts to promote CBE proficiency and the dissemination of breast cancer awareness and evaluation in the remote districts of this mountainous region. The host team included AKHSTj staff and FMDs and FMNs from each district.

## METHODS

The overall aim of our current effort was to increase breast cancer awareness throughout GBAO and for CBE to became a formal exam for all women older than age 35 years who visit family medicine clinics in the districts. After the initial rollout of the CBE screening and training campaign,^5^ we developed a strategy to promote breast health beyond the principal city of Khorog. To accomplish this, we introduced the concept of CBE master trainers (CBE-MTs), who received additional CBE training and mentoring and demonstrated proficiency in the CBE technique and teaching.^5^ To enable the introduction of CBE into the districts, the CBE-MT subsequently conducted trainings with FMNs from each of the districts. In turn, these FMNs, independently or in conjunction with the district FMDs, conducted CBE screening drives in each of their respective districts. All CBEs were conducted in the various family medicine clinics as part of the breast cancer awareness program. All women who presented for CBE provided informed consent.^5^ Women with an abnormal CBE as identified by the FMN and/or FMD were subsequently re-examined by a visiting CBE-MT. Women with confirmed abnormal CBE were referred to the Khorog Regional Diagnostic Unit for diagnostic evaluation according to our previously described CBE algorithm^5^ and referred for additional diagnostic evaluation and treatment as appropriate. Data from each of the districts were collected and verified by AKHSTj staff.

## RESULTS

During the inaugural rollout of the program in 2014, we provided hands-on CBE training to 28 FMDs and seven FMNs. Four hundred forty-one women from the community underwent CBE, and 74 women (17%) had an abnormal exam and underwent diagnostic evaluation. Of these, six patients (1.4% of total screened) were diagnosed with locally advanced breast cancer and received treatment as part of this campaign. The work reporting this result has been previously published.^5^

In 2015, after the initial training, 80 female staff members from the local Aga Khan Development Network units were offered CBE by the CBE-MT. Fifty women consented and underwent CBE. Seven women (14%) had abnormal CBE, but no breast pathology was detected on follow-up diagnostic evaluation in this young patient population, all of whom were younger than age 40 years.

During subsequent visits in 2015 and 2016, 34 FMDs and 18 FMNs received didactic and hands-on CBE training from AKHB-USA clinicians. Some of the participating FMDs and FMNs were part of the original group in 2014 and thus underwent retraining. Overall, a total of 42 FMDs—representing 85% of the FMDs in GBAO—underwent CBE training. During these visits, six CBE-MTs were selected and given additional mentored training.

In 2016, these six CBE-MTs, in turn, independently provided CBE training to 147 FMNs from the districts. Thus, in aggregate, a total of 166 FMNs—representing 55% of the FMNs in GBAO—received CBE training, with some participating in more than one training session. These trained FMNs went on to conduct breast screening campaigns in their respective districts.

In the process, over a 30-month period, a total of 3,556 women in GBAO have undergone CBE. Of these, 696 women (20% of all screened) were found to have an abnormal CBE, and breast cancer was identified in 18 women—2.6% of abnormal CBEs or 0.5% of all screened women.

Table 1 lists the CBE capacity and the community screening accomplished as an outcome of this ongoing collaborative partnership since inception in 2014.

**Table 1:**
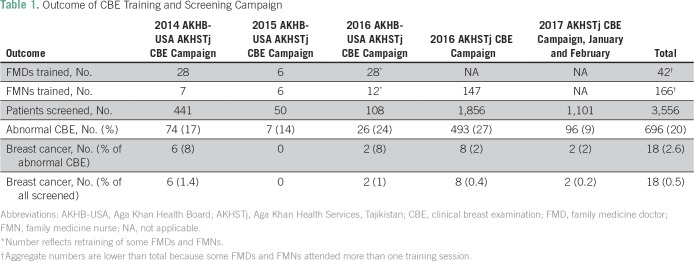
Outcome of CBE Training and Screening Campaign

Whereas the six patients in the original 2014 CBE campaign all had locally advanced breast cancer (clinical stage III or greater), nine of 10 patients who were diagnosed during the 2016 to 2017 screening campaign had locoregional disease (clinical stage II).

Of note, 1,101 women were independently screened by FMNs in the district between January and February 2017, identifying 96 women (9.5%) with an initial abnormal CBE, who were then re-examined by CBE-MTs. Subsequent diagnostic evaluation revealed two women with localized breast cancer (0.2% of all screened women).

## DISCUSSION

With a rise in noncommunicable disease in LMICs^6^ and a focus on women’s health, breast cancer deserves heightened attention in all LMICs, given its prevalence and impact on the quality of life of the individual and family unit. The common occurrence of late presentation and advanced stage in LMICs compared with UMICs^7^ contributes to morbidity, a substantial loss of productive life for patients and productive capacity within the community, and, ultimately, to increased breast cancer mortality.^6,7^

Data that support the benefits of early detection stems from UMICs,^7-10^ where resources and capabilities seem to be limitless. Thus, only one in seven women (15%) in UMICs presents with locally advanced or metastatic breast cancer.^11^ This is in contrast to LMICs, where nearly two of three women have locally advanced or metastatic disease at the time of presentation.^7^ In the context of LMICs, where diagnostic and treatment resources are limited, and with no public health policy that advocates early detection, downstaging of breast cancer at the time of initial detection offers a pragmatic approach to impact morbidity, quality of life, and survival.^7,12-15^ A critical prerequisite for downstaging at presentation is breast cancer awareness on the part of women in the community and health care providers, the training of health care providers in CBE technique, and the promotion of breast health as part of women’s health in the clinical setting.^16-18^

Our efforts were directed at bringing awareness of breast cancer and training the local clinicians—FMDs and FMNs—in the early detection of breast abnormality in the context of promoting CBE and breast health throughout this mountainous region, where access to health care faces socioeconomic hurdles as well as those of climate and terrain. In this context, the AKHSTj has established a remarkable model infrastructure with which to provide basic health resources in each of the mountainous districts through their system of tier 1 district clinics supported by FMNs and visiting FMDs as well as by a network of community lay volunteers.

We began with training FMDs and FMNs in CBE and conducting initial CBE drives in the Oblast capital in Khorog.^5^ The success of this inaugural program was evident by the participation of more than 400 women, the training of 35 health care providers, and the identification of six patients with breast cancer, all of whom received appropriate treatment, established a supportive, trusting environment and the motivation to continue the collaborative effort. In total, 42 FMDs (85% of all FMDs in GBAO) and 166 FMNs (55% of FMNs in GBAO) have since received CBE training as part of this project. Of importance, a majority of the FMNs (147 of 166) received their CBE training from the six CBE-MTs and have gone on to provide the bulk of the CBE screening in the remote district locations. Thus, the biggest incremental increase in CBE training and women screened occurred as a result of the efforts of the trained and experienced local CBE-MTs. Consequently, nearly 1,100 women underwent CBE in the districts over a 2-month period in 2017 compared with 2,400 as part of the original screening drives in Khorugh during the previous 2-year period.

Our data demonstrate a consistent improvement in CBE proficiency by FMDs and FMNs as they became more experienced at performing CBEs (Table 1). Thus, 14% to 27% of women who were screened between 2014 and 2016 had what were characterized as abnormal exams compared with 9% during the most recent CBE campaign in 2017. The screening campaigns often generated great community interest and participation, and many younger women in childbearing age present for CBE. Thus, the fluctuating frequency of abnormal CBE (27% to 9%) may reflect the initial lack of experience and proficiency on the part of clinicians, as well as the palpation of normal, age-related, benign findings in young women who present for screening.^19^

The identification of approximately 1% breast cancer (0.2% to 1.4% of all screened women; Table 1) during our community CBE screening campaign is comparable to that which has been described by others,^20-24^ including findings from a large community-based, clustered randomized control study using trained health care workers in India.^20^ In general, one in 10 women who present at community screening campaigns will have an abnormal CBE, and approximately 1% of the screened population will be diagnosed with breast cancer. These rates may often fluctuate depending on the age of the screened population and the proficiency of the examiner.

Over a 3-year period, we trained (or retrained) a majority (85%) of the FMDs and nearly one half of FMNs in GBAO. The identification of a cadre of CBE-MTs from within the FMDs served to promote the process of ongoing retraining and adjudication of abnormal exams and reflects a major success of the program, as it assures the sustainability of our strategy and an internal self-renewal process for the training of new clinicians as well as the ongoing training/monitoring of trained professionals. The engagement of FMNs from the district assures the promotion of breast health throughout this remote region and underscores the value of the AKHSTj network. Over time, we hope to expand the pool of CBE-MTs and train one FMN master trainer for each district site.

Our program served to establish a network of health care professionals who are equipped to perform CBEs, provided a mechanism for ongoing training and the adjudication of abnormal CBEs, and contributed to the earlier identification of women with abnormal breast masses, thereby contributing to the earlier identification of breast cancer. In the absence of surgical staging data for all women who were diagnosed with breast cancer as part of our campaign, and given the relatively recent implementation of this program, we are unable to determine the impact of our program on the downstaging of breast cancer at time of identification.^20,25^ However, we observed that fewer women with clinical locally advanced tumor masses (clinical stage III or greater) were identified during our subsequent campaigns in 2016 and 2017 compared with our initial campaign in 2014. Thus, all six women with breast cancer who were identified in our original campaign in 2014 had clinical locally advanced stage III or greater disease,^5^ whereas only one of 10 women who were identified during the subsequent campaigns had clinical advanced stage disease. This was an elderly woman who had known about her breast mass for more than 1 year, but had declined intervention, yet was encouraged by her family to participate in the screening campaign in 2016. The patient went on to undergo a palliative mastectomy for her T4 breast tumor. The awareness on the part of the family and the trusting relationship that was established between the CBE team and the community played an important role in encouraging such patients to undergo screening. As part of this project, we identified the need for an experienced psychosocial counselor to provide support to the patient and family in response to the diagnosis of cancer in a cultural environment in which cancer remains stigmatized.

**Fig 2:**
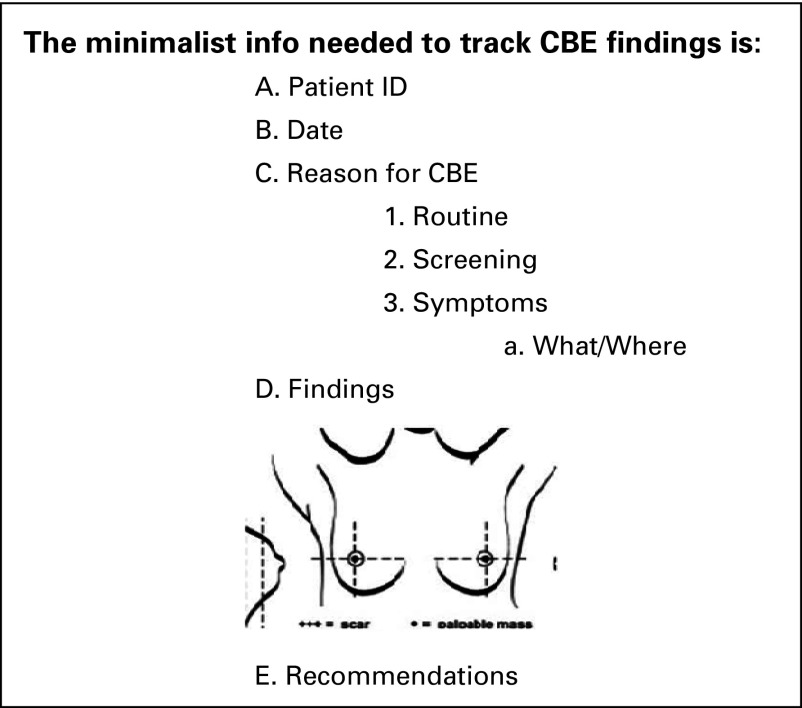
Clinical breast examination (CBE) documentation form.

A key accomplishment of our joint AKHB–AKHSTj program was the adoption of CBE as a routine part of a woman’s exam by the FMD at the Khorog Family Medicine Clinic. Whereas most women attended this clinic for routine ailments, the practice of including CBE provided both experience for the FMD and an opportunity to promote breast health in the female population. Key to sustaining our efforts will be this hands-on practice and an ongoing campaign to incorporate CBE in the general female examination, a practice change that will require endorsement by the health authorities. In addition, we are working with AKHSTj and the health department to advocate for the documentation of CBE findings using a simple form designed as part of our campaign. This form would be completed by the examiner and placed in the patient’s chart for future reference (Fig 2). This is especially relevant for the observation of women with nonspecific abnormal CBE findings living in remote districts, where mammography and/or ultrasound resources are not readily available and travel to the diagnostic center is not feasible or affordable.

Most women in the distant villages may not be able to afford or may lack the resources to travel to the regional center for diagnostic imaging, especially during the harsh winter that lasts nearly 5 months and where there is no access for motorized transportation. Even during the normal season, travel through the mountainous terrain is often on foot or by animal cart. Documentation of the findings on the CBE form (Fig 2) would help to monitor patients with identified CBE abnormality, allow follow-up even when health care providers change, and allow the provider to alert the patient and family if a follow-up CBE reveals objectively documented signs of progression of the abnormal finding.

In 2014, during our initial campaign in Khorog, we were able to collect demographic and clinical information from all patients.^5^ Subsequent CBE campaigns in the district were conducted without the collection of such data. The introduction of CBE documentation and nominal data collection is an important goal, but will require input from the health authorities.

Our project, conducted over 3 years, demonstrates not only the feasibility, but the sustainability, of this effort through a dedicated public-private partnership and a commitment to improving breast cancer awareness within the community and breast health within the medical systems. The success of this program, to date, has been the result of a focused approach on community awareness; identification and mentored training of CBE-MTs, and sustainable training/retraining of FMDs and FMNs; a uniform, algorithm-based approach for the diagnostic evaluation of abnormal findings; and initial efforts at incorporating CBE in routine women’s clinical evaluation. Furthermore, the involvement of FMNs at the forefront of the CBE campaign has ensured the dissemination of the program throughout the Oblast, with FMDs and CBE-MTs often serving as the second tier for evaluation. This strategy has allowed CBE services to be provided to women who live away from the major center and contribute to creating an Oblast-wide awareness of the importance of breast health.

Ongoing monitoring and re-evaluation of the program will be critical to ensuring its long-term success, which can then be linked to the enhancement of diagnostic and treatment services as more patients with cancer are discovered and the demand for advanced breast health services increases. Such efforts will also need to be accompanied by the introduction of clinical documentation and data collection as part of this effort. Although our program shows evidence of traction within the local health care community, the institutionalization of CBE within the health care system, financial coverage of diagnostic studies and treatment that are often unaffordable to many patients, and the negative stigma linked to the diagnosis of breast cancer in the community will need to be addressed strategically if we are to succeed in promoting overall breast health in the region. Ultimately, it will take health policy directives to provide the institutional imperative to support these lifesaving initiatives. Our study shows that strategically designed, goal-oriented public-private partnership focused on training of local resources can be transformative in LMICs in tackling the increasing burden of noncommunicable disease, specifically breast cancer.
